# Role of metabolomic profile as a potential marker to discriminate membranous nephropathy from IgA nephropathy

**DOI:** 10.1007/s11255-023-03691-1

**Published:** 2023-07-15

**Authors:** Yuchen Qu, Yueyuan Wang, Zhanhong Hu, Cunjin Su, Chenyue Qian, Jie Pan, Ye Zhu, Aiming Shi

**Affiliations:** 1 Department of Pharmacy, The Second Affiliated Hospital of Soochow University, Suzhou, Jiangsu China; 2 Department of Nephrology, The Second Affiliated Hospital of Soochow University, Suzhou, Jiangsu China

**Keywords:** Membranous nephropathy, IgA nephropathy, Metabolomics, Receiver operating characteristic (ROC) curve

## Abstract

**Background:**

Membranous nephropathy (MN) and IgA nephropathy (IgAN) are the most common primary glomerulopathies worldwide. The systemic metabolic changes in the progression of MN and IgAN are not fully understood.

**Methods:**

A total of 87 and 70 patients with MN and IgAN, respectively, and 30 healthy controls were enrolled in this study. Untargeted metabolomics was performed to explore the differential metabolites and metabolic pathways in the early stage of MN and IgAN. To judge the diagnostic ability of biomarkers, receiver operating characteristic curve analysis (ROC) were performed.

**Results:**

Principal component analysis (PCA) and orthogonal partial least-squares discriminant analysis (OPLS-DA) suggested that patients with MN and IgAN showed an obvious separation trend from the healthy controls. In addition, 155 and 148 metabolites were identified to be significantly altered in the MN and IgAN groups, respectively. Of these, 70 metabolites were markedly altered in both disease groups; six metabolites, including L-tryptophan, L-kynurenine, gamma-aminobutyric acid (GABA), indoleacetaldehyde, 5-hydroxyindoleacetylglycine, and N-alpha-acetyllysine, showed the opposite tendency. The most affected metabolic pathways included the amino acid metabolic pathways, citrate cycle, pantothenate and CoA biosynthesis, and hormone signaling pathways.

**Conclusions:**

Substantial metabolic disorders occurred during the progression of MN and IgAN. L-tryptophan, L-kynurenine, GABA, indoleacetaldehyde, 5-hydroxyindoleacetylglycine, and N-alpha-acetyllysine may show potential as biomarkers for the identification of MN and IgAN.

**Supplementary Information:**

The online version contains supplementary material available at 10.1007/s11255-023-03691-1.

## Introduction

Chronic kidney disease (CKD) is one of the leading causes of death worldwide. In China, the incidence of CKD has reached 10.8%, and the number is still rapidly growing [1]. Primary glomerular diseases, including membranous nephropathy (MN) and IgA nephropathy (IgAN), are among the leading causes of CKD [2]. Early stages of MN and IgAN typically exhibit an asymptomatic onset with nephrotic-range proteinuria, hypoalbuminemia, edema, and hyperlipidemia. However, these symptoms are neither specific nor sensitive enough for early diagnosis of primary glomerulopathy and still require histopathological evaluation by kidney biopsy [3]. To better determine disease diagnosis and guide the course of treatment, clarifying disease-related metabolic changes and screening potential biomarkers are crucial.

The rapid development of high-throughput technologies, including high-resolution mass spectrometry, sequencing technologies, and microarray, has significantly advanced our understanding of the discovery of biomarkers for primary glomerulopathy. Metabolomics is a newly emerging field of omics research that aims to study global metabolic metabolites in biological systems [4]. In general, the expression of upstream genes and proteins can be amplified by downstream metabolite signals, which makes it easier to detect differences by analyzing metabolite profiling [5]. Therefore, we used a UPLC-Q/Orbitrap-HRMS-based untargeted metabolomics to systematically explore the differential metabolites and metabolic pathways in the early stage of MN and IgAN compared with the healthy population. Furthermore, receiver operating characteristic (ROC) curve analysis was used to judge the diagnostic ability of potential biomarkers.

## Methods

### Study population

From March 2018 to September 2020, 87 and 70 patients with biopsy-proven primary idiopathic MN and biopsy-proven IgAN, respectively, at the Second Affiliated Hospital of Soochow University were enrolled in this study. Renal specimens were evaluated using direct immunofluorescence, light microscopy, and electron microscopy. Patients with secondary forms of glomerulopathy, including hepatitis B viral infection, lupus nephritis, and tumor, were excluded. Only angiotensin receptor blockers (ARBs) were given to a subset of patients before serum sample collection for reducing urine protein excretion. None of these patients received immunosuppressive medications or other therapeutic agents before the diagnosis was established. The corresponding immunosuppressive agents, including methylprednisolone, cyclophosphamide, tripterygium, tacrolimus, cyclosporin, and leflunomide, were administered immediately after the diagnosis was established. Moreover, 30 healthy adult volunteers (HC group) were enrolled in this metabolomics study. This study was approved by the Ethics Committee of the Second Affiliated Hospital of Soochow University.

### Sample collection, preparation, and UPLC-Q/Orbitrap-HRMS analysis

To prepare the serum, 5 mL of fasting blood samples collected from the patients and healthy adult volunteers were centrifuged at 2,500 g for 10 min at 4 °C. All serum samples were immediately stored in a freezer at − 80 °C for further processing.

Next, 400 µL of methanol was added to 100 µL of serum sample in an EP tube. The mixture was vortexed for 1 min and centrifuged at 15,000 g for 10 min at 4 °C. The supernatant was subsequently transferred into another EP tube and evaporated to dry using a vacuum centrifugal concentrator. The residue was resuspended in 150 μL of 80% methanol and filtered through a 0.22-µm nylon syringe filter.

The separation of the target compounds was performed on a Waters ACQUITY UPLC HSS T3 (2.1 × 150 mm, 1.8 µm) liquid chromatography column at 40 °C with an ACQUITY UPLC CSH C18 VanGuard Pre-column (2.1 × 5 mm, 1.7 µm) using a Dionex Ultimate 3000 UPLC system. In positive ion mode, the mobile phase contained 0.1% aqueous formic acid and 0.1% formic acid in acetonitrile. In negative ion mode, the mobile phase contained a 5-mM ammonium formate aqueous buffer and acetonitrile. The mobile phase flow rate was 0.25 mL/min, and the injection volume was 5 µL in both positive and negative ion modes. The Q Exactive Orbitrap mass spectrometer (Thermo Fisher Scientific, USA) equipped with an ESI interface was applied for mass spectrometry analysis. The detailed gradient elution conditions and the optimal mass spectrometry parameters were the same as previously described [6]. All mass spectrometry spectra were acquired and analyzed using the Xcalibur 4.0 software (Thermo Fisher Scientific).

### Data processing and metabolite identification

The data processing and metabolite identification progress was performed as described in our previous study [6]. After the raw data files were converted into an mzXML format using the ProteoWizard software (v3.0.8789), the freely available XCMS software was used to perform peak identification, filtration, alignment, and integration. The three-dimensional data matrix, including retention time, mass-to-charge ratio, and intensity, was converted into a table for further process analysis. To compare the data of different magnitudes, the peak area of the data was batch normalized before multivariate statistical analysis. Subsequently, to perform principal component analysis (PCA) and orthogonal partial least squares discriminant analysis (OPLS-DA), the data were uploaded into SIMCA-P 14.0. Autoscaling was used in all the models to acquire more scientific, reliable, and intuitive results. PCA analysis is an unsupervised multivariate statistical method that can reflect the original state of metabolomic data. OPLS-DA is a multivariate statistical analysis method with supervised pattern recognition, which can effectively propose the effects unrelated to the study, to screen the differential metabolites. To select the potential metabolites in the study, the variable importance in the project values (VIP) of the OPLS-DA model and *p* values were calculated as a threshold value (VIP > 1 combined with *p* < 0.05). These potential metabolites were subsequently subjected to pathway analysis performed through METLIN (http://metlin.scripps.edu/), MoNA (https://mona.fiehnlab.ucdavis.edu/), and BioDeepDB (https://query.biodeep.cn/). To elucidate metabolic differences between the MN and IgAN groups, the identified metabolites were compared. Moreover, to judge the diagnostic ability of biomarkers, ROC curve analyses were performed using MetaboAnalyst 5.0 (https://www.metaboanalyst.ca/).

## Results

### Clinicopathological characteristics of patients

In this study, 87 and 70 patients with MN and IgAN, respectively, and 30 healthy adult volunteers were enrolled to determine metabolic changes in the progression of MN and IgAN. The baseline characteristics of the healthy volunteers and the patients before renal biopsies are presented in Table 1. The pathological features of patients are shown in Supplementary Material.

**Table 1:** Baseline characteristics of the patients and the healthy volunteers

Characteristics	MN (*n* = 87)	IgAN (*n* = 70)	HC (*n* = 30)
Age, year	52.0, 42.0–62.0	35.0, 31.0–43.8	50.0, 44.3–63.8
Gender, male:female	52:35	37:33	15:15
Body mass index, kg/m^2^	24.3, 22.5–27.0	24.2, 22.4–26.1	24.0, 22.3–24.9
Serum creatinine, μmol/L	66.0, 53.5–79.0	92.5, 65.8–116.3	61.0, 55.0–69.8
Serum albumin, g/L	25.6, 20.9–29.4	37.7, 34.4–41.8	45.5, 43.8–47.1
Proteinuria, g/24 h	4.9, 3.1–7.4	2.0, 1.3–3.1	–
Total cholesterol, mmol/L	7.0,5.6–8.7	4.9, 4.4–5.7	4.5, 4.0–4.9
Serum uric acid, mg/dL	6.2, 5.1–7.2	6.5, 5.5–7.8	3.6, 3.0–4.1
Urea nitrogen, mmol/L	5.3, 4.4–6.2	5.8, 4.6–6.8	4.7, 4.1–5.1
Triglyceride, mmol/L	2.2, 1.4–2.9	1.5, 1.2–2.2	1.1, 0.8–1.4
Hypertension, *n* (%)	23 (26.4%)	25 (35.7%)	–
Diabetes, *n* (%)	9 (10.3%)	3 (4.3%)	–

Continuous variables are expressed as medians (quartiles), and categorical variables are described as frequencies (percentages)

### Multivariate statistical analysis

**Fig. 1:**
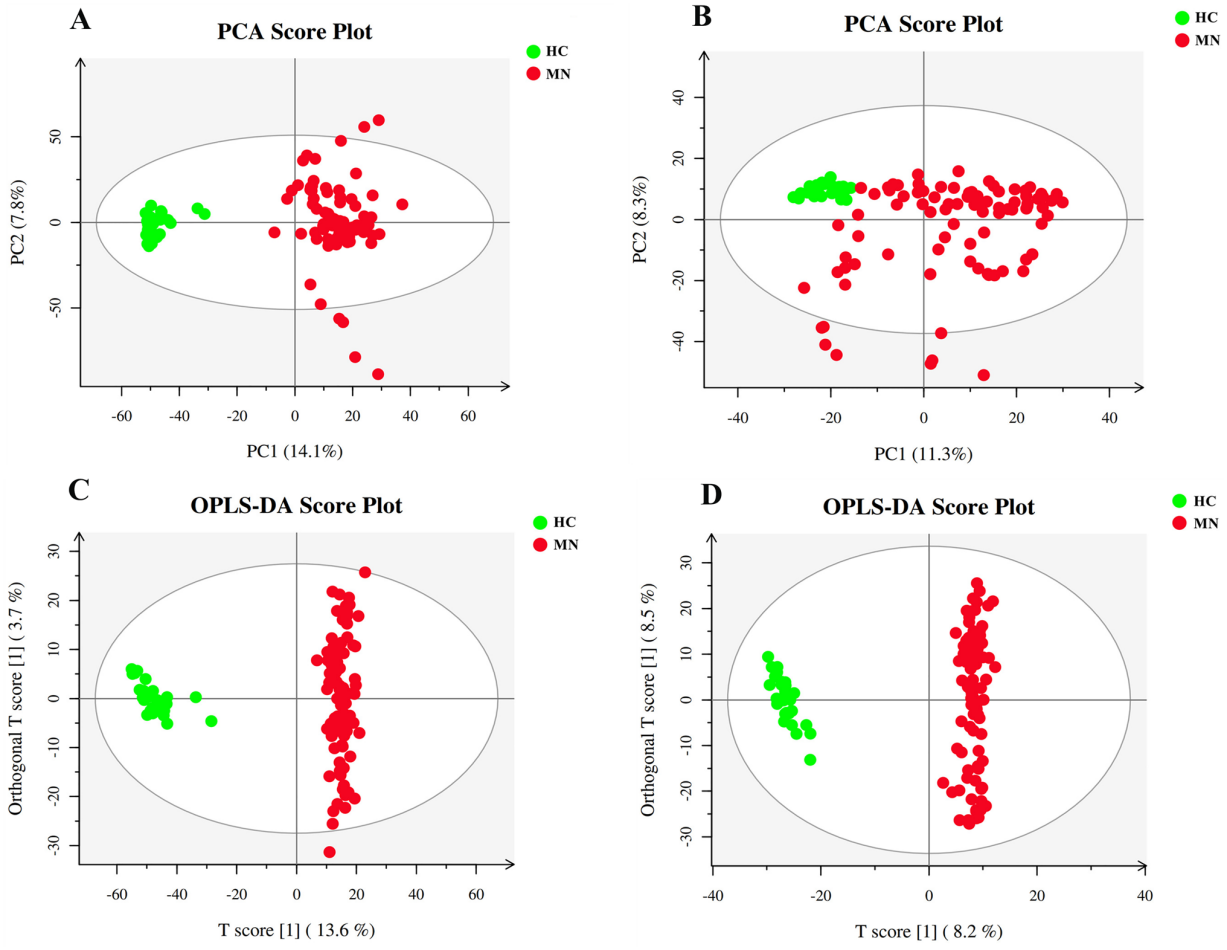
Multivariate data analysis of the membranous nephropathy (MN) and healthy control (HC) groups. **A** Principal component analysis (PCA) score map for the positive ion mode, **B** PCA score map for the negative ion mode, **C** Orthogonal partial least squares discriminant analysis (OPLS-DA) score map for the positive ion mode, and **D** OPLS-DA score map for the negative ion mode

**Fig. 2:**
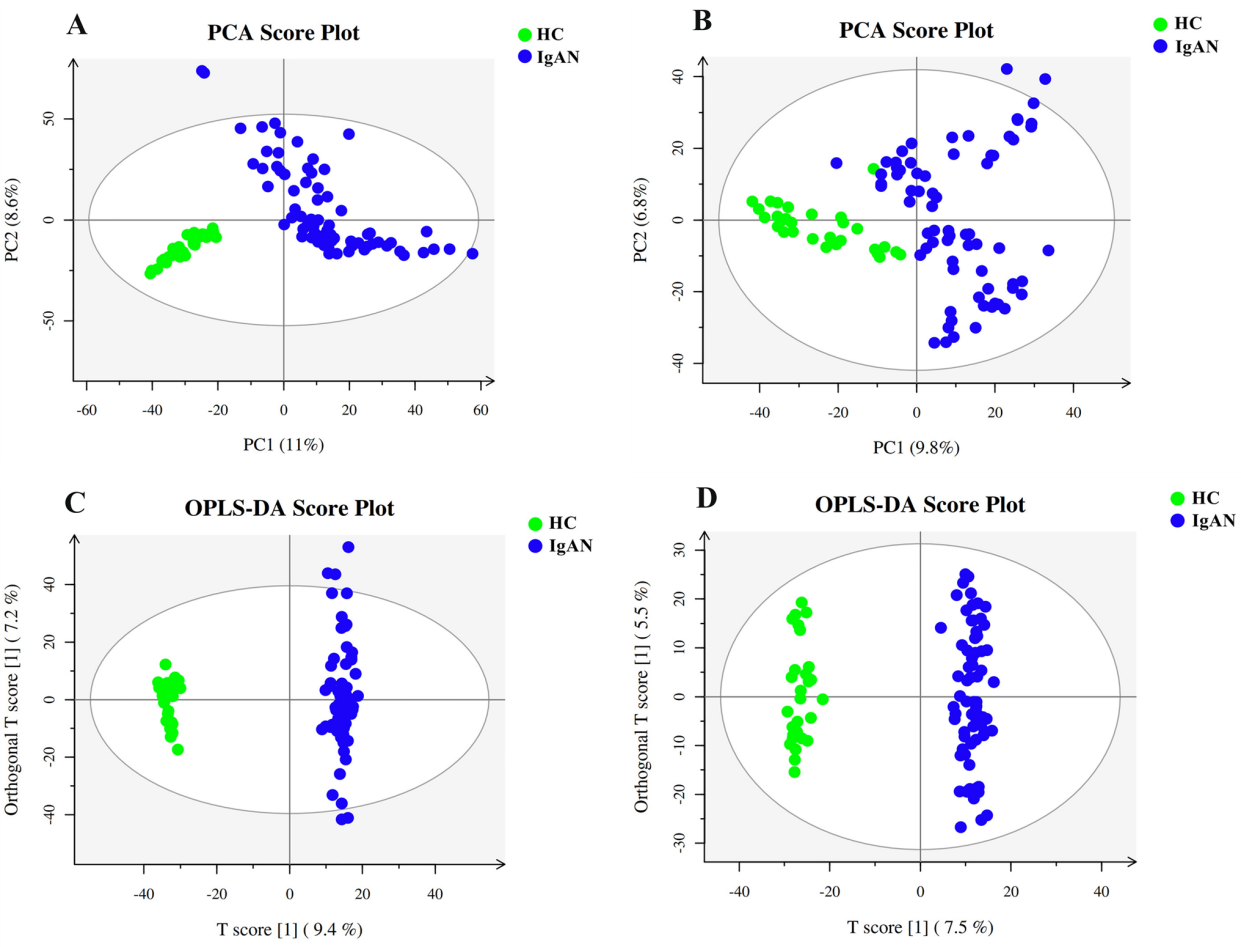
Multivariate data analysis of the IgA nephropathy (IgAN) and healthy control (HC) groups. **A** PCA score map for the positive ion mode, **B** PCA score map for the negative ion mode, **C** OPLS-DA score map for the positive ion mode, and **D** OPLS-DA score map for the negative ion mode

To screen for the specific metabolites in the development and progression of idiopathic MN, PCA and OPLS-DA were used for grasping the overall situation and identifying inter-group differences in the data, respectively. As shown in Figs. 1 and 2, the MN and IgAN groups showed an obvious separation trend from the healthy controls. The following were the R^2^Y (represents the seed setting rate) and Q^2^ (represents the prediction ability of the model) values of the OPLS-DA model in this study: MN/HC-ESI(+), R^2^Y = 0.980, Q^2^ = 0.956; MN/HC-ESI(−), R^2^Y = 0.987, Q^2^ = 0.944; IgAN/HC-ESI( +), R^2^Y = 0.993, Q^2^ = 0.961; IgAN/HC-ESI(−), R^2^Y = 0.987, Q^2^ = 0.920. These results showed that the models had a strong explanatory ability and could be reliably used to screen for differential metabolites.

### Metabolite screening in untargeted metabolomics analysis

Using VIP > 1.0 and *p* < 0.05 as the thresholds in our study, 155 and 148 metabolites were identified to be significantly altered in the MN and IgAN groups, respectively. In detail, 71 metabolites were significantly increased and 84 metabolites were significantly decreased in the MN group, whereas 85 metabolites were significantly increased and 63 metabolites were significantly decreased in the IgAN group. Furthermore, the results indicated that 70 metabolites were markedly altered in both disease groups. Detailed information on these metabolites is shown in Table 2.

**Table 2:** Identification of different metabolites

Name	KEGG ID	Molecular formula	Class	Pathway	MN/HC	IgAN/HC
Hydrogen phosphate	C00009	H3O4P	Non-metal oxoanionic compounds	Oxidative phosphorylation; parathyroid hormone synthesis, secretion and action	↑	↑
Pyridoxal phosphate	C00018	C8H10NO6P	Organoheterocyclic compounds	Vitamin B6 metabolism; thiamine metabolism	↓	↓
S-Adenosylhomocysteine	C00021	C14H20N6O5S	Lactones	Cysteine and methionine metabolism	–	↓
Pyruvic acid	C00022	C3H4O3	Keto acids and derivatives	Citrate cycle	↓	–
l-Glutamic acid	C00025	C5H9NO4	Carboxylic acids and derivatives	Multiple amino acid metabolism pathways	↑	↑
Succinic acid	C00042	C4H6O4	Carboxylic acids and derivatives	Citrate cycle	↓	–
Sulfate	C00059	H2O4S	Non-metal oxoanionic compounds	Sulfur metabolism; purine metabolism; cysteine and methionine metabolism	↑	–
l-Arginine	C00062	C6H14N4O2	Carboxylic acids and derivatives	Arginine and proline metabolism	–	↓
l-Tryptophan	C00078	C11H12N2O2	Indoles and derivatives	Multiple amino acid metabolism pathways	↓	↑
l-Phenylalanine	C00079	C9H11NO2	Carboxylic acids and derivatives	Phenylalanine metabolism	↓	–
l-Tyrosine	C00082	C9H11NO3	Carboxylic acids and derivatives	Tyrosine metabolism	↓	–
Glucose 6-phosphate	C00092	C6H13O9P	Organooxygen compounds	Inositol phosphate metabolism; starch and sucrose metabolism	–	↑
l-Cysteine	C00097	C3H7NO2S	Carboxylic acids and derivatives	Cysteine and methionine metabolism; pantothenate and CoA biosynthesis	↓	–
Uracil	C00106	C4H4N2O2	Diazines	Pantothenate and CoA biosynthesis; pyrimidine metabolism	–	↓
2-Ketobutyric acid	C00109	C4H6O3	Keto acids and derivatives	Multiple amino acid metabolism pathways; propanoate metabolism	–	↓
Dihydroxyacetone phosphate	C00111	C3H7O6P	Organooxygen compounds	Nicotinate and nicotinamide metabolism; multiple carbohydrate metabolism-related pathways	↓	↓
Choline	C00114	C5H14NO	Organonitrogen compounds	Glycerophospholipid metabolism	–	↑
Biotin	C00120	C10H16N2O3S	Biotin and derivatives	Vitamin digestion and absorption; biotin metabolism	↑	–
Fumaric acid	C00122	C4H4O4	Carboxylic acids and derivatives	Citrate cycle	↓	↓
l-Leucine	C00123	C6H13NO2	Carboxylic acids and derivatives	Valine, leucine and isoleucine biosynthesis and degradation	↑	↑
Putrescine	C00134	C4H12N2	Organonitrogen compound	Multiple amino acid metabolism pathways	↑	–
Myo-Inositol	C00137	C6H12O6	Organooxygen compounds	Galactose metabolism; inositol phosphate metabolism; ascorbate and aldarate metabolism	↑	↑
N-Acetyl-D-glucosamine	C00140	C8H15NO6	Organooxygen compounds	Amino sugar and nucleotide sugar metabolism	–	↓
alpha-Ketoisovaleric acid	C00141	C5H8O3	Keto acids and derivatives	Valine, leucine and isoleucine biosynthesis and degradation; pantothenate and CoA biosynthesis	↓	↓
GMP	C00144	C10H14N5O8P	Purine nucleotides	Purine metabolism	↓	–
l-Proline	C00148	C5H9NO2	Carboxylic acids and derivatives	Arginine and proline metabolism	–	↑
l-Asparagine	C00152	C4H8N2O3	Carboxylic acids and derivatives	Alanine, aspartate and glutamate metabolism	–	↓
Niacinamide	C00153	C6H6N2O	Pyridines and derivatives	Nicotinate and nicotinamide metabolism	–	↑
4-Hydroxybenzoic acid	C00156	C7H6O3	Benzene and substituted derivatives	Ubiquinone and other terpenoid-quinone biosynthesis	↓	–
Citric acid	C00158	C6H8O7	Carboxylic acids and derivatives	Citrate cycle	↓	↓
d-Mannose	C00159	C6H12O6	Organooxygen compounds	NA	–	↓
Acetoacetic acid	C00164	C4H6O3	Keto acids and derivatives	Multiple amino acid metabolism pathways; ketone body biosynthesis	↓	↓
Phenylpyruvic acid	C00166	C9H8O3	Benzene and substituted derivatives	Phenylalanine metabolism	–	↓
Thymine	C00178	C5H6N2O2	Diazines	Pyrimidine metabolism	↓	–
d-Xylose	C00181	C5H10O5	Organooxygen compounds	Amino sugar and nucleotide sugar metabolism	↑	–
l-Valine	C00183	C5H11NO2	Carboxylic acids and derivatives	Valine, leucine and isoleucine biosynthesis and degradation; pantothenate and CoA biosynthesis	↑	↑
d-Ribulose 5-phosphate	C00199	C5H11O8P	Organooxygen compounds	Pentose phosphate pathway; riboflavin metabolism	–	↑
Adenosine	C00212	C10H13N5O4	Purine nucleosides	Purine metabolism; sphingolipid signaling pathway	–	↑
Thymidine	C00214	C10H14N2O5	Pyrimidine nucleosides	Pyrimidine metabolism	–	↓
Arachidonic acid	C00219	C20H32O2	Fatty Acyls	Arachidonic acid metabolism	↑	–
d-Glucose	C00221	C6H12O6	Organooxygen compounds	Glycolysis/gluconeogenesis; pentose phosphate pathway	↑	↑
Succinic acid semialdehyde	C00232	C4H6O3	Fatty Acyls	Nicotinate and nicotinamide metabolism	–	↑
Ketoleucine	C00233	C6H10O3	Keto acids and derivatives	Valine, leucine and isoleucine biosynthesis and degradation	–	↓
Guanine	C00242	C5H5N5O	Imidazopyrimidines	Purine metabolism	–	↑
Palmitic acid	C00249	C16H32O2	Fatty Acyls	Fatty acid and unsaturated fatty acids biosynthesis	↑	–
Hypoxanthine	C00262	C5H4N4O	Imidazopyrimidines	Purine metabolism	↑	–
2,3-Butanediol	C00265	C4H10O2S2	Organooxygen compounds	NA	↑	–
Androstenedione	C00280	C19H26O2	Steroids and steroid derivatives	Steroid hormone biosynthesis	↑	↑
Inosine	C00294	C10H12N4O5	Purine nucleosides	Purine metabolism	↑	↑
Uridine	C00299	C9H12N2O6	Pyrimidine nucleosides	Pyrimidine metabolism	↓	–
Creatine	C00300	C4H9N3O2	Carboxylic acids and derivatives	Arginine and proline metabolism; glycine, serine and threonine metabolism	↑	–
Spermidine	C00315	C7H19N3	Organonitrogen compounds	Arginine and proline metabolism	↓	–
Oxoadipic acid	C00322	C6H8O5	Keto acids and derivatives	Tryptophan metabolism; lysine degradation	↓	–
Citrulline	C00327	C6H13N3O3	Carboxylic acids and derivatives	Arginine biosynthesis	–	↑
l-Kynurenine	C00328	C10H12N2O3	Organooxygen compounds	Tryptophan metabolism	↓	↑
gamma-Aminobutyric acid	C00334	C4H9NO2	Carboxylic acids and derivatives	Multiple amino acid metabolism pathways; butanoate metabolism; nicotinate and nicotinamide metabolism	↑	↓
l-Dopa	C00355	C9H11NO4	Carboxylic acids and derivative	Tyrosine metabolism	–	↓
dAMP	C00360	C10H14N5O6P	Purine nucleotides	Purine metabolism	↓	–
Uric acid	C00366	C5H4N4O3	Imidazopyrimidines	Purine metabolism	↑	↑
d-Xylitol	C00379	C5H12O5	Organooxygen compounds	Pentose and glucuronate interconversions	↑	↑
Cytosine	C00380	C4H5N3O	Diazines	Pyrimidine metabolism	↓	–
Malonate	C00383	C3H4O4	Fatty acyls	Pyrimidine metabolism; fatty acid biosynthesis	–	↓
Guanosine	C00387	C10H13N5O5	Purine nucleosides	Purine metabolism	–	↑
Mannitol	C00392	C6H14O6	Organooxygen compounds	Fructose and mannose metabolism	↓	–
Tryptamine	C00398	C10H12N2	Indoles and derivatives	Tryptophan metabolism	–	↑
Ubiquinone-1	C00399	C14H18O4	Prenol lipids	Ubiquinone and other terpenoid-quinone biosynthesis	↓	–
l-Isoleucine	C00407	C6H13NO2	Carboxylic acids and derivatives	Valine, leucine and isoleucine biosynthesis and degradation	–	↑
cis-Aconitic acid	C00417	C6H6O6	Carboxylic acids and derivatives	Citrate cycle	↑	–
trans-Cinnamate	C00423	C9H8O2	Cinnamic acids and derivatives	Phenylalanine metabolism; ubiquinone and other terpenoid-quinone biosynthesis	↑	↑
Dihydrouracil	C00429	C4H6N2O2	Diazines	Pantothenate and CoA biosynthesis; pyrimidine metabolism	–	↑
5-Aminopentanoic acid	C00431	C5H11NO2	Carboxylic acids and derivatives	Lysine degradation	–	↓
N-Acetylornithine	C00437	C7H14N2O3	Carboxylic acids and derivatives	Arginine biosynthesis	–	↓
Formiminoglutamic acid	C00439	C6H10N2O4	Carboxylic acids and derivatives	Histidine metabolism	↓	–
Indole	C00463	C8H7N	Indoles and derivatives	Tryptophan metabolism	↓	–
Ribitol	C00474	C5H12O5	Organooxygen compounds	Riboflavin metabolism	↓	–
Ecdysone	C00477	C27H44O6	Steroids and steroid derivatives	NA	↑	↑
Tyramine	C00483	C8H11NO	Benzene and substituted derivatives	Tyrosine metabolism	–	↓
l-Cystine	C00491	C6H12N2O4S2	Carboxylic acids and derivatives	Cysteine and methionine metabolism	–	↓
Shikimic acid	C00493	C7H10O5	Organooxygen compounds	Phenylalanine, tyrosine and tryptophan biosynthesis	–	↓
Erythritol	C00503	C4H10O4	Organooxygen compounds	NA	↓	↓
d-Ornithine	C00515	C5H12N2O2	Carboxylic acids and derivatives	D-Arginine and D-ornithine metabolism	↑	–
Pyridoxamine	C00534	C8H12N2O2	Pyridines and derivatives	Vitamin B6 metabolism	–	↑
Benzoate	C00539	C7H6O2	Benzene and substituted derivatives	NA	↑	↑
Homogentisic acid	C00544	C8H8O4	Benzene and substituted derivatives	Tyrosine metabolism; ubiquinone and other terpenoid-quinone biosynthesis	–	↓
Norepinephrine	C00547	C8H11NO3	Phenols	Tyrosine metabolism	↑	↑
Deoxyadenosine	C00559	C10H13N5O3	Purine nucleosides	Purine metabolism	↓	–
Betaine aldehyde	C00576	C5H12NO	Organonitrogen compounds	Glycine, serine and threonine metabolism	↓	–
Guanidinoacetate	C00581	C3H7N3O2	Carboxylic acids and derivatives	Arginine and proline metabolism; glycine, serine and threonine metabolism	–	↓
Phenylacetaldehyde	C00601	C8H8O	Benzene and substituted derivatives	Phenylalanine metabolism	↓	–
N-Acetylglutamic acid	C00624	C7H11NO5	Carboxylic acids and derivatives	Arginine biosynthesis	↓	↓
Gentisic acid	C00628	C7H6O4	Benzene and substituted derivatives	Tyrosine metabolism	↓	↓
3-Hydroxyanthranilic acid	C00632	C7H7NO3	Benzene and substituted derivatives	Tryptophan metabolism	↓	–
Indoleacetaldehyde	C00637	C10H9NO	Indoles and derivatives	Tryptophan metabolism	↓	↑
gamma-Glutamylcysteine	C00669	C8H14N2O5S	Carboxylic acids and derivatives	Glutathione metabolism	↓	↓
Betaine	C00719	C5H12NO2	Carboxylic acids and derivatives	Glycine, serine and threonine metabolism	–	↑
Vanillin	C00755	C8H8O3	Phenols	NA	↓	–
Creatinine	C00791	C4H7N3O	Carboxylic acids and derivatives	Arginine and proline metabolism	–	↑
Salicylic acid	C00805	C7H6O3	Benzene and substituted derivatives	Phenylalanine metabolism	↑	–
Glucaric acid	C00818	C6H10O8	Organooxygen compounds	Ascorbate and aldarate metabolism	↓	–
1-Hexadecanol	C00823	C16H34O	Fatty Acyls	Fatty acid degradation	↑	↑
l-Arogenate	C00826	C10H13NO5	Carboxylic acids and derivatives	Phenylalanine, tyrosine and tryptophan biosynthesis	↑	–
4-Pyridoxic acid	C00847	C8H9NO4	Pyridines and derivatives	Vitamin B6 metabolism	↑	↑
l-Histidinol	C00860	C6H11N3O	Organonitrogen compounds	Histidine metabolism	↑	↑
Pantothenic acid	C00864	C9H17NO5	Organooxygen compounds	Pantothenate and CoA biosynthesis	↑	↑
Porphobilinogen	C00931	C10H14N2O4	Organonitrogen compounds	Porphyrin metabolism	↓	↓
Cyclic GMP	C00942	C10H12N5O7P	Purine nucleotides	Purine metabolism	↓	–
Estradiol	C00951	C18H24O2	Steroids and steroid derivatives	Steroid hormone biosynthesis	↑	–
Indole-3-acetate	C00954	C10H9NO2	Indoles and derivatives	Tryptophan metabolism	↓	–
Trigonelline	C01004	C7H7NO2	Alkaloids	Nicotinate and nicotinamide metabolism	–	↑
6-Hydroxynicotinate	C01020	C6H5NO3	Pyridines and derivatives	Nicotinate and nicotinamide metabolism	↑	–
4-Guanidinobutanoic acid	C01035	C5H11N3O2	Carboxylic acids and derivatives	Arginine and proline metabolism	↓	↓
Ascorbate	C01041	C6H8O6	Dihydrofurans	Glutathione metabolism; ascorbate and aldarate metabolism	↑	↑
3-Hydroxybutyric acid	C01089	C4H8O3	Hydroxy acids and derivatives	Butanoate metabolism	–	↓
Sphinganine 1-phosphate	C01120	C18H40NO5P	Sphingolipids	Sphingolipid metabolism	↓	↓
1-Methylhistidine	C01152	C7H11N3O2	Carboxylic acids and derivatives	Histidine metabolism	–	↑
cis-4-Hydroxy-D-proline	C01157	C5H9NO3	Carboxylic acids and derivatives	Arginine and proline metabolism	–	↑
4-Hydroxyphenylpyruvic acid	C01179	C9H8O4	Benzene and substituted derivatives	Tyrosine metabolism; ubiquinone and other terpenoid-quinone biosynthesis	↑	–
3-(2-Hydroxyphenyl)propanoic acid	C01198	C9H10O3	Phenylpropanoic acids	Phenylalanine metabolism	–	↓
Dehydroepiandrosterone	C01227	C19H28O2	Steroids and steroid derivatives	Steroid hormone biosynthesis	↓	–
l-Iditol	C01507	C6H14O6	Organooxygen compounds	NA	↓	–
Hippuric acid	C01586	C9H9NO3	Benzene and substituted derivatives	Phenylalanine metabolism	↓	↓
Threonic acid	C01620	C4H8O5	Organooxygen compounds	Ascorbate and aldarate metabolism	↓	↓
Calcitriol	C01673	C27H44O3	Steroids and steroid derivatives	Mineral absorption; parathyroid hormone synthesis, secretion and action	↓	–
Galactitol	C01697	C6H14O6	Organooxygen compounds	Galactose metabolism	–	↓
Mesaconate	C01732	C5H6O4	Fatty Acyls	Glyoxylate and dicarboxylate metabolism	–	↑
Pyroglutamic acid	C01879	C5H7NO3	Carboxylic acids and derivatives	Glutathione metabolism	–	↓
d-Arabitol	C01904	C5H12O5	Organooxygen compounds	Pentose and glucuronate interconversions	↑	↑
l-Histidinal	C01929	C6H9N3O	Organonitrogen compounds	Histidine metabolism	↑	↑
(R)-mandelic Acid	C01983	C8H8O3	Benzene and substituted derivatives	NA	↓	–
Acetylcholine	C01996	C7H16NO2	Organonitrogen compounds	Glycerophospholipid metabolism	–	↑
Glycyl-glycine	C02037	C4H8N2O3	Carboxylic acids and derivatives	NA	–	↑
Indolelactic acid	C02043	C11H11NO3	Indoles and derivatives	NA	↓	↓
Pseudouridine	C02067	C9H12N2O6	Nucleoside and nucleotide analogues	Pyrimidine metabolism	–	↑
Citraconic acid	C02226	C5H6O4	Fatty Acyls	Valine, leucine and isoleucine biosynthesis	↑	–
d-Fructose	C02336	C6H12O6	Organooxygen compounds	Amino sugar and nucleotide sugar metabolism	↓	–
Beta-D-Fructose	C02336	C6H12O6	Organooxygen compounds	Amino sugar and nucleotide sugar metabolism	–	↓
N-Methyltyramine	C02442	C9H13NO	Benzene and substituted derivatives	Tyrosine metabolism	↑	↑
Xanthurenic acid	C02470	C10H7NO4	Quinolines and derivatives	Tryptophan metabolism	↓	↓
2-Isopropylmalic acid	C02504	C7H12O5	Fatty Acyls	Valine, leucine and isoleucine biosynthesis	↑	↑
O-Acetylcarnitine	C02571	C9H18NO4	Fatty Acyls	Insulin resistance	↑	–
Isobutyric acid	C02632	C4H8O2	Carboxylic acids and derivatives	NA	–	↑
3-Dehydroshikimate	C02637	C7H7O5	Organooxygen compounds	Phenylalanine, tyrosine and tryptophan biosynthesis	↑	–
Pimelic acid	C02656	C7H12O4	Fatty Acyls	Biotin metabolism	↑	↑
d-Glucurono-6,3-lactone	C02670	C6H8O6	Furofurans	Ascorbate and aldarate metabolism	↑	–
N6-Acetyl-L-lysine	C02727	C8H16N2O3	Carboxylic acids and derivatives	Lysine degradation	–	↑
Hydroxykynurenine	C02794	C10H12N2O4	Organooxygen compounds	NA	↓	–
3-Indoleacetonitrile	C02938	C10H8N2	Indoles and derivatives	Tryptophan metabolism	↑	↑
3-Dehydro-L-threonate	C03064	C4H6O5	Ascorbate metabolic products	Ascorbate and aldarate metabolism	–	↓
DL-Glycerol 1-phosphate	C03189	C3H9O6P	Glycerophospholipids	NA	↑	–
2-Keto-6-aminocaproate	C03239	C6H11NO3	Keto acids and derivatives	Lysine degradation	–	↑
Imidazol-5-yl-pyruvate	C03277	C6H6N2O3	Histidine metabolic products	Histidine metabolism	–	↓
Decanoyl-L-carnitine	C03299	C17H33NO4	Fatty Acyls	NA	↓	↓
N-Acetyl-L-phenylalanine	C03519	C11H13NO3	Carboxylic acids and derivatives	Phenylalanine metabolism	–	↓
Ciliatine	C03557	C2H8NO3P	Organic phosphonic acids and derivatives	Phosphonate and phosphinate metabolism	↓	–
Asymmetric dimethylarginine	C03626	C8H18N4O2	Carboxylic acids and derivatives	NA	↑	↑
(-)-Bornesitol	C03659	C7H14O6	Organooxygen compounds	Inositol phosphate metabolism	↑	↑
Quinolinic acid	C03722	C7H5NO4	Pyridines and derivatives	Tryptophan metabolism; nicotinate and nicotinamide metabolism	↓	–
Dopamine	C03758	C8H11NO2	Phenols	Tyrosine metabolism	–	↑
4-Hydroxyphenylacetaldehyde	C03765	C8H8O2	Benzene and substituted derivatives	Tyrosine metabolism	–	↑
2-Oxoarginine	C03771	C6H11N3O3	Keto acids and derivatives	D-Arginine and D-ornithine metabolism	↓	–
Dihydrotestosterone	C03917	C19H30O2	Steroids and steroid derivatives	Steroid hormone biosynthesis	↑	–
LysoPA(16:0/0:0)	C04036	C19H39O7P	Glycerophospholipids	NA	–	↓
Bovinic acid	C04056	C18H32O2	Fatty Acyls	Linoleic acid metabolism	–	↑
3D-3,5/4-Trihydroxycyclohexane-1,2-dione	C04287	C6H8O5	myo-Inositol catabolism derivatives	Inositol phosphate metabolism	↓	–
13-L-Hydroperoxylinoleic acid	C04717	C18H32O4	Fatty Acyls	Linoleic acid metabolism	–	↓
13(S)-HPOT	C04785	C18H30O4	Fatty Acyls	alpha-Linolenic acid metabolism	↓	–
7-Dehydrodesmosterol	C05107	C27H42O	Steroids and steroid derivatives	Steroid biosynthesis	↑	↑
(2R,5S)-2,5-Diaminohexanoate	C05161	C6H14N2O2	Lysine metabolic products	Lysine degradation	–	↑
Phenylethylamine	C05332	C8H11N	Benzene and substituted derivatives	Phenylalanine metabolism	↓	↓
Fructose 1,6-bisphosphate	C05378	C6H14O12P2	Organooxygen compounds	Multiple carbohydrate metabolism-related pathways	↓	–
Sedoheptulose 7-phosphate	C05382	C7H15O10P	Organooxygen compounds	Pentose phosphate pathway	–	↑
Ergosta-5,7,22,24(28)-tetraen-3beta-ol	C05440	C28H42O	Sterol lipids	Steroid biosynthesis	–	↓
Deoxyinosine	C05512	C10H12N4O4	Purine nucleosides	Purine metabolism	–	↑
Beta-D-3-Ribofuranosyluric acid	C05513	C10H12N4O7	Imidazopyrimidines	Purine metabolism	–	↑
2-Keto-6-acetamidocaproate	C05548	C8H13NO4	Keto acids and derivatives	Lysine degradation	↓	↓
3,4-Dihydroxyphenylglycol	C05576	C8H10O4	Phenols	Tyrosine metabolism	↓	–
Homovanillin	C05581	C9H10O3	Phenols	Tyrosine metabolism	↓	–
Homovanillic acid	C05582	C9H10O4	Phenols	Tyrosine metabolism	↑	↑
Vanillylmandelic acid	C05584	C9H10O5	Phenols	Tyrosine metabolism	↓	–
Vanylglycol	C05594	C9H12O4	Phenols	Tyrosine metabolism	↑	↑
D-Phenyllactic acid	C05607	C9H10O3	Phenylpropanoic acids	Phenylalanine metabolism	–	↑
Hydrocinnamic acid	C05629	C9H10O2	Phenylpropanoic acids	Phenylalanine metabolism	↑	–
Tetrahydropteridine	C05650	C6H8N4	Pteridines and derivatives	NA	↓	–
Formylanthranilic acid	C05653	C8H7NO3	Benzene and substituted derivatives	Tryptophan metabolism	–	↓
Methylimidazoleacetic acid	C05828	C6H8N2O2	Azoles	Histidine metabolism	↑	↑
5-Hydroxyindoleacetylglycine	C05832	C12H12N2O4	Carboxylic acids and derivatives	Tryptophan metabolism	↓	↑
2-Hydroxyphenylacetate	C05852	C8H8O3	Benzene and substituted derivatives	Phenylalanine metabolism	↓	↓
2-Phenylethanol	C05853	C8H10O	Benzene and substituted derivatives	Phenylalanine metabolism	↑	↑
2-Oxo-4-hydroxy-5-aminovalerate	C05941	C5H9NO4	Proline metabolic products	Arginine and proline metabolism	↑	–
Pyrrole-2-carboxylic acid	C05942	C5H5NO2	Pyrroles	Arginine and proline metabolism	–	↓
Tyrosol	C06044	C8H10O2	Phenols	Tyrosine metabolism	↑	↑
Sphingosine 1-phosphate	C06124	C18H38NO5P	Sphingolipids	Sphingolipid metabolism	↓	–
Caprylic acid	C06423	C8H16O2	Fatty Acyls	Lipoic acid metabolism	–	↑
Myristic acid	C06424	C14H28O2	Fatty Acyls	Fatty acid biosynthesis	↓	–
Diethylphosphate	C06608	C4H11O4P	Organic phosphoric acids and derivatives	NA	↑	–
4-Methylcatechol	C06730	C7H8O2	Phenols	NA	↑	↑
Epiandrosterone	C07635	C19H30O2	Steroids and steroid derivatives	NA	–	↓
Azelaic acid	C08261	C9H16O4	Fatty Acyls	NA	–	↑
3-Methylthiopropionic acid	C08276	C4H8O2S	Fatty Acyls	Cysteine and methionine metabolism; sulfur metabolism	–	↓
Sebacic acid	C08277	C10H18O4	Fatty Acyls	NA	–	↑
Suberic acid	C08278	C8H14O4	Fatty Acyls	NA	↑	↑
3-Methylindole	C08313	C9H9N	Indoles and derivatives	Tryptophan metabolism	↓	–
Skatole	C08313	C9H9N	Indoles and derivatives	Tryptophan metabolism	–	↑
Tetracosanoic acid	C08320	C24H48O2	Fatty Acyls	Biosynthesis of unsaturated fatty acids	↓	↓
Indican	C08481	C14H17NO6	Organooxygen compounds	NA	↓	–
Jasmonic acid	C08491	C12H18O3	Fatty Acyls	alpha-Linolenic acid metabolism	↓	–
Benzamide	C09815	C7H7NO	Benzene and substituted derivatives	NA	–	↓
Eugenol	C10453	C10H12O2	Phenols	NA	↑	↑
2,6-Dimethoxyphenol	C10787	C8H10O3	Phenols	NA	↑	↑
Dimethyl sulfone	C11142	C2H6O2S	Sulfonyls	Sulfur metabolism	↑	↑
(S)-1-Phenylethanol	C11348	C8H10O	Benzene and substituted derivative	NA	↑	–
N-alpha-acetyllysine	C12989	C8H16N2O3	Carboxylic acids and derivatives	NA	↓	↑
1-Arachidonoylglycerol	C13857	C23H38O4	Glycerolipids	NA	–	↑
Equol	C14131	C15H14O3	Isoflavonoids	NA	↓	–
13S-Hydroxyoctadecadienoic acid	C14762	C18H32O3	Fatty Acyls	Linoleic acid metabolism, PAR signaling pathway	–	↓
9-OxoODE	C14766	C18H30O3	Fatty Acyls	Linoleic acid metabolism	↓	–
8,9-EET	C14769	C20H32O3	Fatty Acyls	Arachidonic acid metabolism	↑	↑
8,9-DiHETrE	C14773	C20H34O4	Fatty Acyls	Arachidonic acid metabolism	↓	↓
9(S)-HPODE	C14827	C18H32O4	Fatty Acyls	Linoleic acid metabolism	–	↓
9,10-DHOME	C14828	C18H34O4	Fatty Acyls	Linoleic acid metabolism	↓	–
9,10-Dihydroxy-12,13-epoxyoctadecanoate	C14837	C18H34O5	Fatty acyls	Linoleic acid metabolism	↓	–
25-Hydroxycholesterol	C15519	C27H46O2	Steroids and steroid derivatives	Primary bile acid biosynthesis	–	↑
N-a-Acetylcitrulline	C15532	C8H15N3O4	Carboxylic acids and derivative	Arginine biosynthesis	↑	–
Benzylamine	C15562	C7H9N	Benzene and substituted derivatives	NA	↑	–
Phenyl acetate	C15583	C8H8O2	Phenol esters	NA	↓	↓
Traumatic Acid	C16308	C12H20O4	Fatty Acyls	Alpha-Linolenic acid metabolism	↑	↑
Dehypoxanthine futalosine	C17010	C14H16O7	Intermediates in menaquinone biosynthesis	Ubiquinone and other terpenoid-quinone biosynthesis	↑	–
Heptanoic acid	C17714	C7H14O2	Fatty Acyls	NA	↓	–
Undecanoic acid	C17715	C11H22O2	Fatty Acyls	NA	–	↓
N-Acetyldemethylphosphinothricin	C17949	C6H12NO5P	Intermediates in phosphinate metabolism	Phosphonate and phosphinate metabolism	↓	–
Ethylmethylacetic acid	C18319	C5H10O2	Fatty Acyls	Protein digestion and absorption	↓	–
Hexadecanedioate	C19615	C16H30O4	Fatty Acyls	NA	↑	–
(R)-3-Ureidoisobutyrate	C21029	C5H10N2O3	Organic carbonic acids and derivatives	Pyrimidine metabolism	↓	–

### Metabolic pathway analysis

**Fig. 3:**
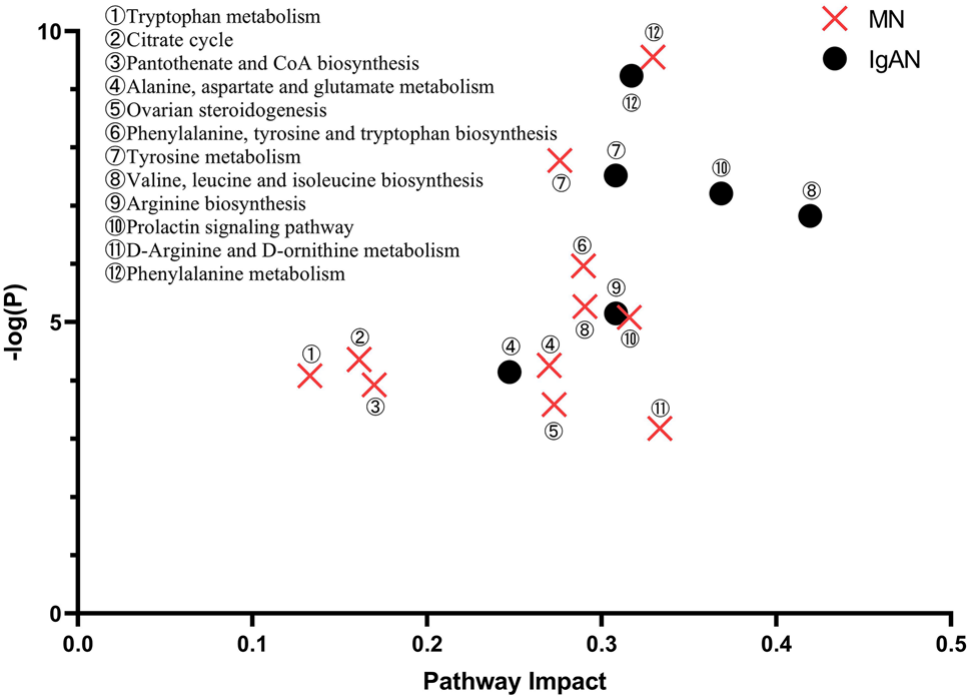
Statistically significant differential pathways (*p* < 0.05) identified in the MN and IgAN groups

To further search for the metabolic pathways that potential biomarkers may participate in, MetaboAnalyst 5.0 was used to analyze the potential metabolite data obtained above. As shown in Fig. 3, a total of 11 pathways were identified to be statistically significant (*p* < 0.05), which may be potentially associated with MN, including (1) phenylalanine metabolism; (2) tyrosine metabolism; (3) phenylalanine, tyrosine, and tryptophan biosynthesis; (4) valine, leucine, and isoleucine biosynthesis; (5) prolactin signaling pathway; (6) citrate cycle; (7) arginine and proline metabolism; (8) tryptophan metabolism; (9) pantothenate and CoA biosynthesis; (10) ovarian steroidogenesis; and (11) D-arginine and D-ornithine metabolism. Meanwhile, six pathways were identified to be statistically significant in the IgAN group, including (1) phenylalanine metabolism; (2) tyrosine metabolism; (3) valine, leucine, and isoleucine biosynthesis; (4) prolactin signaling pathway; (5) arginine and proline metabolism; and (6) arginine biosynthesis.

### Identification of potential biomarkers for MN and IgAN

**Fig. 4:**
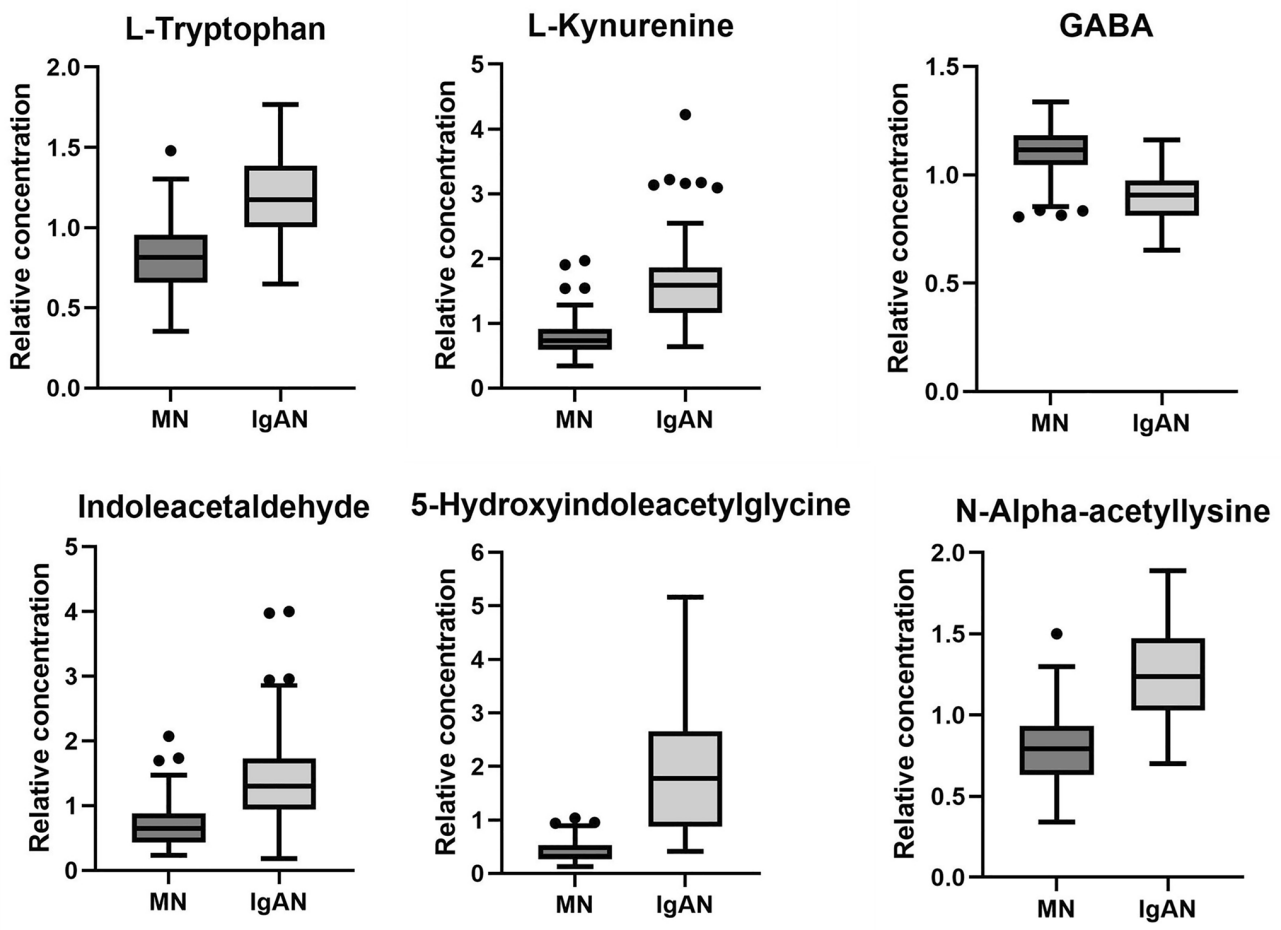
Boxplot of six potential biomarkers between the MN and IgAN groups. Peak areas are normalized to the corresponding mean peak area of the HC, which is defined as 1

**Fig. 5:**
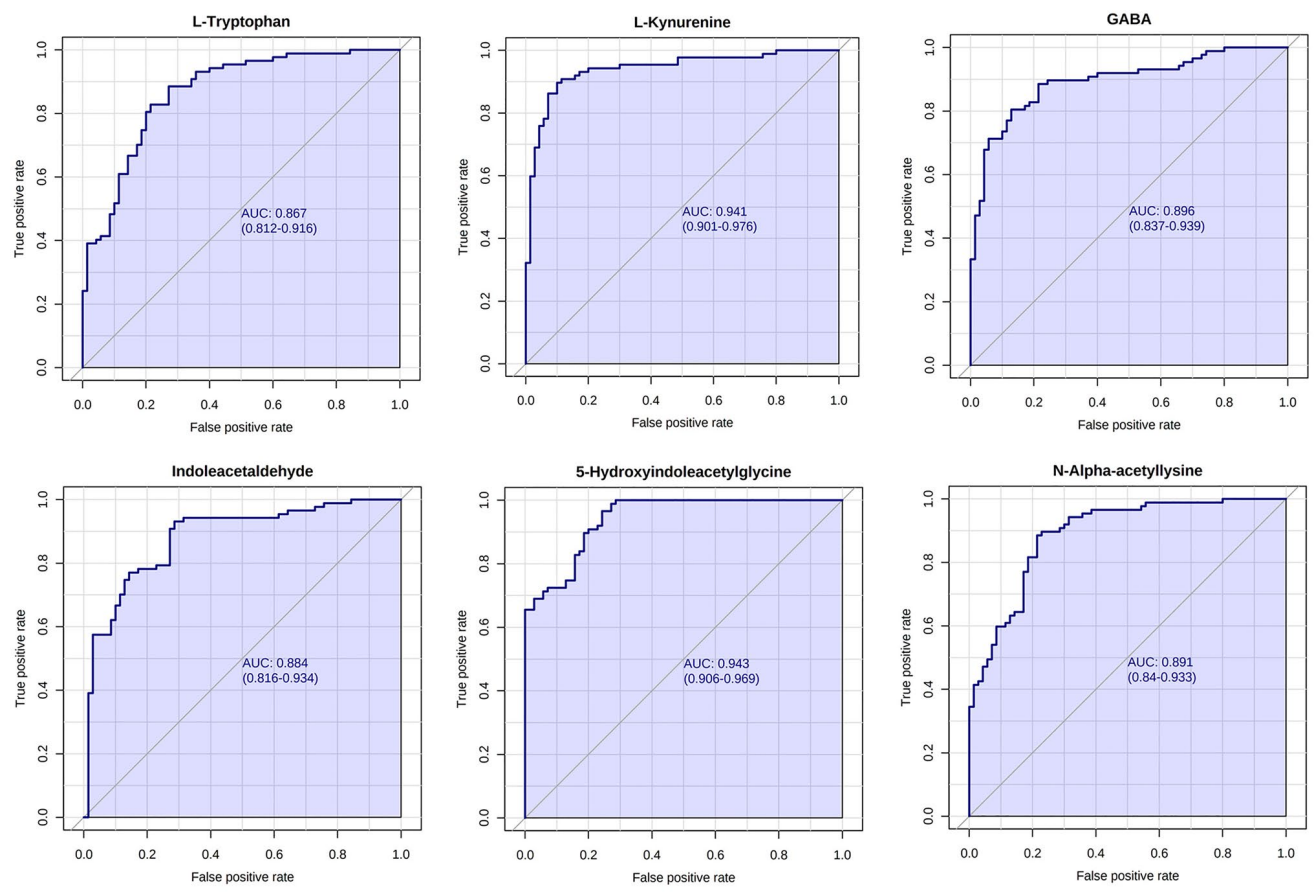
Receiver operating characteristic curve analysis for the six potential biomarkers of the MN and IgAN groups

Six metabolites exerted the opposite tendency in the MN and IgAN groups, including L-tryptophan, L-kynurenine, GABA, indoleacetaldehyde, 5-hydroxyindoleacetylglycine, and N-alpha-acetyllysine (Fig. 4). The values of the area under the ROC curve were 0.867, 0.941, 0.896, 0.884, 0.943, and 0.891, respectively, suggesting the high predictive performance of these metabolites for the identification of MN and IgAN (Fig. 5).

## Discussion

Metabolomics has become an important diagnostic tool that can directly reflect the state of organisms [7]. Compared with conventional identification methods, such as biopsy, metabolomics has the advantage that the contents of various metabolites can be analyzed and distinguished from disease populations [8]. To investigate the metabolic changes during the progression of MN and IgAN, untargeted metabolomics research was first performed. The results showed that 155 and 148 metabolites were identified to be significantly altered in the MN and IgAN groups, respectively. Through further metabolic pathway analysis, we observed that 11 and 6 metabolic pathways were significantly affected in the patients with MN and IgAN, including amino acid metabolic pathways, citrate cycle, pantothenate and CoA biosynthesis, and hormone signaling pathway. Here, we attempted to provide a detailed discussion on these affected metabolic pathways and compare the metabolic differences between the MN and IgAN groups.

Substantial metabolic disorders occurred during the progression of primary glomerulopathy; according to the metabolomics findings, amino acid metabolic pathways were the most affected. In humans, the kidneys play a significant role in the metabolism and reabsorption of amino acids. In our study, in the MN group, the relative contents of L-glutamic acid, L-valine, and L-leucine significantly increased, whereas those of L-cysteine, L-tryptophan, L-phenylalanine, and L-tyrosine significantly decreased. In the IgAN group, the relative contents of L-glutamic acid, L-valine, L-leucine, L-isoleucine, L-tryptophan, and L-proline significantly increased, whereas those of L-arginine, L-asparagine, and L-cysteine significantly decreased. The abnormal metabolism of amino acids may be caused by the increase in protein-decomposing metabolism or the change of energy demand in the inflammatory state. Therefore, the patients should acquire a low-protein diet containing sufficient essential amino acids, which can improve the imbalance of amino acid metabolism and subsequently increase protein synthesis.

The tricarboxylic acid (TCA) cycle is a common metabolic pathway in organisms, which is performed in the mitochondria of eukaryotic cells. It is not only the final metabolic pathway of the three nutrients (e.g., sugars, lipids, and amino acids) but also the link between them [9]. In this study, the relative contents of citric, fumaric, and succinic acids in the MN and IgAN groups decreased, suggesting that the TCA cycle is decelerated in patients with MN and IgAN. This result was consistent with the results of a previous study that patients with MN with more impaired kidney filtration function exhibited lower citric acid levels in serum [10]. In addition, the abnormal levels of citric, fumaric, and succinic acids suggest that mitochondrial function is damaged, which indirectly reflects the obvious oxidative stress in patients with MN. This may be the result of renal ischemia and hypoxia-induced cell necrosis, which is consistent with the previous results reported in vitro [11, 12].

Furthermore, our results showed that pantothenate and CoA biosynthesis was significantly affected during the progression of MN. Pantothenate is an essential vitamin as it is the pivotal precursor of CoA, which plays an essential role in the TCA cycle and energy metabolism [13]. The pantothenate and CoA biosynthesis pathway is actively involved in the inflammatory response and host defense by generating cysteamine. Cysteamine can directly inhibit the activity of γ-glutamylcysteine synthase, the rate-limiting enzyme involved in the metabolism from glutamate to γ-glutamy-L-cysteine (the precursor of glutathione) [14]. We observed a significant decrease in the γ-glutamy-L-cysteine level with a significant increase in the glutamate level, suggesting that glutathione biosynthesis was blocked during the progression of MN. Our study indicates that pantothenate and glutathione metabolism may play a significant role in MN development.

Moreover, the kidney is an essential site for the synthesis and degradation of several hormones [15, 16]. We observed that multiple hormone signaling pathways, including the prolactin signaling pathway, glucagon signaling pathway, and ovarian steroidogenesis, may participate in the pathogenesis of endocrine abnormalities in patients with MN and IgAN. The results indicate that various endocrine disorders may have occurred in the early stage of primary glomerulopathy.

In addition to these significantly affected metabolic pathways, the changes of specific metabolites must be paid attention to. We observed that a multitude of gut-derived protein-bound uremic toxins, including hippuric acid, kynurenine, indole-3-acetate, quinolinic acid, and spermidine, were all significantly downregulated in the early stage of MN. Similar results were also obtained in the IgAN group, wherein multiple uremic toxins, including hippuric acid, guanidinoacetate, and N-acetyl-L-phenylalanine, presented a significant decline. Previous studies reported that the accumulation of gut-derived protein-bound uremic toxins in patients with CKD was a critical risk factor for cardiovascular damage [17]. Therefore, we can infer that the disturbed metabolic processes in MN may also dynamically change as the disease develops. The damage caused by the elevated uremic toxin levels in these patients is not alarming, at least in the early stages of disease development. Moreover, our results showed that the levels of pyridoxic and homovanillic acids, which are two novel endogenous serum biomarkers of organic anion transporter (OAT)1 and OAT3, were all significantly upregulated in the MN and IgAN groups [18]. The abovementioned evidence indicated that the decline in the tubular secretion function may be earlier than the decline in the glomerular filtration function. Therefore, the use of drugs that are mainly eliminated by active secretion may deserve more attention in the early stage of primary glomerulopathy.

This study had several limitations. First, differences in the baseline characteristics of the patients in the two groups during sample collection were noted, making disease progression a confounding factor for differential diagnosis between the two groups. We considered propensity score matching for comparison; however, we did not perform it because of the small number of participants after matching. This study may truly differentiate patients with IgAN and primary MN from healthy controls; however, potential biomarkers are yet to be concluded. These biomarkers did not differentiate the aforementioned diseases from other kidney diseases, including CKD and secondary glomerular disease. Therefore, using these metabolites as alone markers might not be specific indicators for the clinical diagnosis. Still, further study will be needed. Furthermore, we were unable to control for diet among all patients, which might have an influence on the energy metabolism pathway and related metabolites.

The most significantly affected pathways were observed to be highly overlapped during the progression of MN and IgAN. To screen for potential biomarkers to distinguish between the MN and IgAN groups, metabolites, including tryptophan, kynurenine, GABA, indoleacetaldehyde, 5-hydroxyindoleacetylglycine, and N-alpha-acetyllysine, were observed to exert opposite tendency in the MN and IgAN groups compared with the healthy controls. The ROC curve analysis confirmed the potential diagnostic value of all six metabolites. Notably, four (e.g., tryptophan, kynurenine, indoleacetaldehyde, and 5-hydroxyindoleacetylglycine) of the six metabolites were associated with the tryptophan metabolism pathway. Further analysis suggested that the kynurenine pathway, the major (95%) catabolic route for tryptophan [19], was significantly downregulated in the MN group. Multiple intermediate or end-product metabolites of kynurenine, including 3-hydroxyanthranilic, quinolinic, and xanthurenic acids, significantly decreased in the MN group. In the IgAN group, apart from the increased tryptophan and kynurenine levels, no notable changes were observed in the levels of the metabolites of kynurenine. Existing studies had indicated that the disorder of tryptophan metabolism in IgAN patients might be associated with the disturbed intestinal microbiome. De Angelis’s study revealed that tryptophan levels increased with progressed disease stage of IgAN [20], which was corroborated with the findings in our study. Wu’s study also revealed that the conversion of tryptophan to kynurenine was enhanced in IgAN patients [21]. They proposed that the accumulation of kynurenine in the circulation system was related to the reduced renal function and the abnormal kynurenine metabolism caused by immune dysregulation and chronic inflammation in the host [21, 22]. It remained unclear how the progression of MN affected tryptophan metabolism. Our results indicated that tryptophan and its metabolites were potential biomarkers for discriminating between IgAN and MN. First, the patients in the IgAN group had worse renal function as compared to the patients in the MN group, which might lead to the reduced glomerular filtration of tryptophan and its metabolites. Meanwhile, there were significant differences in the gut microbial community composition and abundance between IgAN patients and MN patients, which might contribute to the difference in disorder of intestinal tryptophan–indole metabolic pathway [23, 24]. Moreover, considering the importance of the kynurenine pathway for its role in anti-inflammation in kidney disease [25], we inferred that the metabolic differences in this pathway might also be related to the difference in the pathological basis between the two glomerulopathies. However, there is currently scant evidence to support this conclusion. To further investigate the prognostic performance of these potential biomarkers, targeted metabolomics analysis will be performed in our future research.

### Supplementary Information

Below is the link to the electronic supplementary material.

Supplementary file1 (DOCX 36 KB)

## Data Availability

The data sets used during the current study are available from the corresponding author on reasonable request.
