# Supplementary material for: Role of metabolomic profile as a potential marker to discriminate membranous nephropathy from IgA nephropathy
Source: Int Urol Nephrol. 2023 Jul 15;56(2):635–51. doi: 10.1007/s11255-023-03691-1 (PMC10808257; doi:10.1007/s11255-023-03691-1)
Supplement: Supplementary file 1 — Supplementary file1 (DOCX 36 KB) [file 11255_2023_3691_MOESM1_ESM.docx]

**Supplementary Material**

**suppl. Table 1.** Pathological features of patients in membranous nephropathy group

| ID | IgA deposition | IgG deposition | IgM deposition | C3 deposition | C4 deposition | C1q deposition | PLA2R1 | Pathological stage |
| --- | --- | --- | --- | --- | --- | --- | --- | --- |
| MN-1 | - | +++ | - | ++ | ± | - | ++ | II |
| MN-2 | - | +++ | - | + | - | - | ++ | II |
| MN-3 | - | +++ | ± | ++ | - | + | +++ | II |
| MN-4 | - | ++ | - | ++ | - | - | ++ | II |
| MN-5 | - | ++ | - | + | - | - | - | I |
| MN-6 | - | ++ | - | ++ | - | - | ++ | II |
| MN-7 | + | ++ | - | - | - | - | - | II |
| MN-8 | - | +++ | - | - | - | - | ++ | III |
| MN-9 | - | ++ | - | + | - | - | ++ | II/III |
| MN-10 | - | - | - | - | - | - | ++ | II/III |
| MN-11 | - | ++ | - | + | - | ± | ++ | II |
| MN-12 | - | + | - | - | - | - | ++ | I |
| MN-13 | - | +++ | - | + | - | - | ++ | III |
| MN-14 | - | +++ | - | ++ | - | - | ++ | II |
| MN-15 | - | ++ | - | ± | - | - | ++ | I |
| MN-16 | - | ++ | - | + | - | + | ++ | III |
| MN-17 | - | +++ | - | ++ | ± | ± | ++ | II |
| MN-18 | - | ++ | + | ++ | ++ | ± | ++ | II |
| MN-19 | - | - | - | - | - | - | ++ | I |
| MN-20 | - | ++ | - | + | - | - | ++ | I |
| MN-21 | - | ++ | - | + | ++ | - | ++ | I/II |
| MN-22 | - | ++ | - | + | - | - | ++ | II |
| MN-23 | - | + | - | ± | - | - | ++ | I |
| MN-24 | + | +++ | - | + | - | - | ++ | II |
| MN-25 | - | ++ | - | + | - | - | ++ | I |
| MN-26 | - | ++ | - | + | ++ | - | ++ | I |
| MN-27 | - | ++ | - | ++ | ± | - | ++ | II |
| MN-28 | - | ++ | - | ++ | - | - | ++ | II |
| MN-29 | - | - | - | - | - | - | ++ | I |
| MN-30 | - | - | - | - | - | - | +++ | II |
| MN-31 | - | ++ | - | ± | - | - | ++ | I/II |
| MN-32 | - | ++ | - | ++ | - | + | ++ | I |
| MN-33 | - | ± | - | ± | - | - | ++ | II |
| MN-34 | - | +++ | - | ++ | ± | ± | ++ | I |
| MN-35 | - | +++ | - | ++ | - | - | ++ | II |
| MN-36 | - | ++ | - | - | - | - | ++ | I |
| MN-37 | - | ++ | - | + | - | ± | ++ | II |
| MN-38 | - | ++ | - | ± | - | - | ++ | I/II |
| MN-39 | - | ++ | - | + | - | - | + | I/II |
| MN-40 | - | - | - | - | - | - | ++ | I |
| MN-41 | - | - | - | - | - | - | ++ | III |
| MN-42 | - | +++ | - | ++ | - | - | ++ | I |
| MN-43 | + | +++ | - | + | - | ± | +++ | II |
| MN-44 | - | ++ | - | ++ | - | - | ++ | II |
| MN-45 | + | ++ | - | + | ± | + | ++ | III |
| MN-46 | - | ++ | - | - | - | - | ++ | I |
| MN-47 | - | ++ | - | + | - | - | ++ | I |
| MN-48 | - | ++ | + | ± | ± | - | ++ | III |
| MN-49 | - | ++ | - | - | - | - | ± | II |
| MN-50 | - | ++ | - | + | - | - | ++ | II |
| MN-51 | - | ++ | - | + | ± | - | ++ | II |
| MN-52 | - | ++ | - | ++ | - | - | ++ | III |
| MN-53 | - | ++ | - | ++ | - | - | ++ | II |
| MN-54 | - | ++ | - | ± | - | - | ++ | III |
| MN-55 | - | ++ | - | ± | - | - | ++ | II |
| MN-56 | - | ++ | - | + | - | - | ++ | I |
| MN-57 | ++ | +++ | - | ± | - | - | +++ | II |
| MN-58 | - | +++ | + | +++ | + | - | ++ | III |
| MN-59 | - | +++ | - | ++ | - | - | ++ | II |
| MN-60 | - | +++ | - | ++ | - | - | ++ | I |
| MN-61 | ++ | +++ | - | + | - | - | ++ | I |
| MN-62 | + | +++ | - | ++ | - | ± | +++ | III |
| MN-63 | - | ++ | - | + | - | - | ++ | II |
| MN-64 | - | ++ | - | ++ | - | - | ++ | II |
| MN-65 | - | ++ | - | +~++ | - | - | ++ | I/II |
| MN-66 | - | ++ | - | + | - | + | ++ | II |
| MN-67 | + | +++ | - | ++ | - | ± | +++ | III |
| MN-68 | - | +++ | - | ++ | - | ± | ++ | II |
| MN-69 | - | ± | - | ± | - | - | - | IV |
| MN-70 | - | ++ | - | + | - | - | ++ | II |
| MN-71 | - | ++ | - | + | - | - | ++ | I |
| MN-72 | - | ++ | - | - | - | - | - | I/II |
| MN-73 | - | ++ | - | + | - | ± | ++ | II |
| MN-74 | - | +++ | - | +++ | - | - | +++ | I |
| MN-75 | - | ++ | - | ± | - | - | ++ | I |
| MN-76 | - | ++ | - | - | - | - | ++ | I |
| MN-77 | - | ++ | - | - | - | - | ++ | II |
| MN-78 | - | +++ | - | - | - | - | +++ | III |
| MN-79 | + | ± | - | - | - | - | - | I |
| MN-80 | + | ++ | - | ± | - | - | ++ | II |
| MN-81 | - | ++ | - | - | - | - | ++ | III |
| MN-82 | - | ++ | - | + | - | - | ++ | I |
| MN-83 | - | + | - | + | - | - | ++ | II |
| MN-84 | - | ++ | - | ± | - | - | ++ | II |
| MN-85 | - | +~++ | - | ± | - | + | ++ | II |
| MN-86 | - | +++ | - | ++ | + | - | ++ | II |
| MN-87 | - | ++ | - | + | - | - | ++ | I |

**suppl. Table 2.** Pathological features of patients in IgA nephropathy group

| ID | IgA deposition | IgG deposition | IgM deposition | C3 deposition | C4 deposition | C1q deposition | Oxford score |
| --- | --- | --- | --- | --- | --- | --- | --- |
| IgA-1 | ++ | - | - | - | - | - | M0E1S1T0C2 |
| IgA-2 | ++/+++ | - | - | - | - | - | M1E1S1T0C1 |
| IgA-3 | ++ | - | - | - | - | - | M0E0S1T0C1 |
| IgA-4 | +/++ | - | - | - | - | - | M0E1S1T0C0 |
| IgA-5 | ++ | - | - | - | - | - | M1E1S1T1C1 |
| IgA-6 | +++ | - | - | ++ | - | - | M1E0S1T1C1 |
| IgA-7 | ++ | - | - | - | - | - | M0E0S1T0C0 |
| IgA-8 | +/++ | - | - | + | - | - | M0E1S1T0C1 |
| IgA-9 | + | - | - | - | - | - | M0E0S0T0C0 |
| IgA-10 | + | - | - | ± | - | - | M1E0S1T0C1 |
| IgA-11 | +/++ | - | - | - | - | - | M0E0S1T1C0 |
| IgA-12 | +/++ | - | - | +/++ | - | - | M0E1S1T2C1 |
| IgA-13 | ± | - | - | + | - | - | M0E0S1T2C0 |
| IgA-14 | + | - | - | +/++ | - | - | M1E0S1T0C1 |
| IgA-15 | +/++ | - | - | + | - | - | M0E1S1T0C1 |
| IgA-16 | +/++ | - | +/++ | - | - | - | M0E1S1T0C0 |
| IgA-17 | +/++ | - | - | +/++ | - | - | M0E0S1T1 |
| IgA-18 | ++ | + | - | ++ | - | - | M0E1S1T0 |
| IgA-19 | ++/+++ | - | - | +/++ | - | - | M0E0S1T0 |
| IgA-20 | +/++ | - | - | + | - | - | M0E0S1T1 |
| IgA-21 | +/++ | - | - | + | - | - | M0E1S1T0C1 |
| IgA-22 | + | - | - | +/++ | - | - | M0E1S1T0C1 |
| IgA-23 | +++ | - | - | ++ | - | - | M1E0S1T0 |
| IgA-24 | ++ | - | - | + | - | - | M0E0S0T0C0 |
| IgA-25 | +/++ | - | - | + | - | - | M0E1S1T0C0 |
| IgA-26 | ++ | - | - | +/++ | - | - | M0E1S0T0C1 |
| IgA-27 | +/++ | - | - | - | - | - | M0E1S1T1C0 |
| IgA-28 | ++ | - | - | ++ | - | - | M1E1S1T0C1 |
| IgA-29 | ++ | - | - | +/++ | - | - | M0E0S1T0C1 |
| IgA-30 | ++ | - | - | ++ | - | - | M0E0S1T1C0 |
| IgA-31 | +/++ | - | - | + | - | - | M0E0S1T0C1 |
| IgA-32 | ++/+++ | - | - | ± | - | - | M0E1S1T0C0 |
| IgA-33 | ++ | - | - | ± | - | - | M0E0S1T0 |
| IgA-34 | +/++ | - | - | +/++ | - | - | M0E1S1T0C1 |
| IgA-35 | +/++ | - | - | + | - | - | M1E0S1T0C0 |
| IgA-36 | +/++ | - | - | +/++ | - | - | M1E1S1T2C0 |
| IgA-37 | +/++ | - | - | + | - | - | M0E0S0T0C0 |
| IgA-38 | ++ | - | - | + | - | - | M0E1S1T0 |
| IgA-39 | + | - | - | ± | - | - | M0E1S1T1 |
| IgA-40 | ++ | - | - | +/++ | - | - | M0E0S1T2C1 |
| IgA-41 | + | - | - | ± | - | - | M0E0S0T0C0 |
| IgA-42 | ++ | - | - | +/++ | - | - | M0E1S1T0C1 |
| IgA-43 | ++ | - | - | ++ | - | - | M1E0S1T1C0 |
| IgA-44 | +/++ | - | - | +/++ | - | - | M0E1S1T1C1 |
| IgA-45 | ++ | - | - | +/++ | - | - | M0E0S1T0C0 |
| IgA-46 | ++ | - | - | + | - | - | M0E0S1T0 |
| IgA-47 | + | - | - | ± | - | - | M0E1S1T0C1 |
| IgA-48 | + | - | - | + | - | - | M1E0S1T1C0 |
| IgA-49 | +/++ | - | - | +/++ | - | - | M0E0S1T0 |
| IgA-50 | +/++ | - | - | + | - | - | M0E0S1T0C0 |
| IgA-51 | + | - | - | + | - | - | M0E0S0T0C0 |
| IgA-52 | + | - | - | + | - | - | M0E1S1T0C1 |
| IgA-53 | ± | - | - | - | - | - | M0E0S0T0C1 |
| IgA-54 | ++/+++ | - | + | + | - | - | M1E1S1T0 |
| IgA-55 | ++ | - | - | +/++ | - | - | M0E1S1T0 |
| IgA-56 | ++ | - | - | ++ | - | - | M0E1S0T0 |
| IgA-57 | +/++ | - | +/++ | - | - | - | M0E1S1T0C0 |
| IgA-58 | ++ | - | - | + | - | - | M0E0S1T0 |
| IgA-59 | + | - | - | ± | - | - | M1E1S1T1C0 |
| IgA-60 | +/++ | - | - | + | - | - | M0E1S1T0C1 |
| IgA-61 | ++ | - | - | +/++ | - | - | M1E1S1T1 |
| IgA-62 | ++ | + | - | +/++ | - | - | M0E1S1T0 |
| IgA-63 | ++/+++ | - | - | ++/+++ | - | - | M0E1S1T0 |
| IgA-64 | +/++ | - | - | +/++ | - | - | M1E0S1T0 |
| IgA-65 | +/++ | - | - | + | - | - | M1E0S0T0 |
| IgA-66 | +/++ | - | - | - | - | - | M1E0S1T1C0 |
| IgA-67 | ++ | - | - | - | - | - | M1E0S1T0C1 |
| IgA-68 | ++ | - | - | + | - | - | M1E0S1T0C0 |
| IgA-69 | ++/+++ | - | - | ++/+++ | - | - | M0E1S1T0 |
| IgA-70 | + | - | - | + | - | - | M0E0S0T0C1 |
